# Heat Shock Protein 27 in Radiation-Induced Trismus: Mechanistic Insights and a Hypothesis-Generating Framework

**DOI:** 10.3390/biomedicines14051091

**Published:** 2026-05-12

**Authors:** Erkan Topkan, Efsun Somay, Doga Topkan, Sukran Senyurek, Duriye Ozturk, Ugur Selek

**Affiliations:** 1 Department of Radiation Oncology, Faculty of Medicine, Baskent University, Adana 01120, Turkey; docdretopkan@gmail.com; 2 Department of Oral and Maxillofacial Surgery, Faculty of Dentistry, Baskent University, Ankara 06753, Turkey; 3 Department of Clinical Pharmacy, Faculty of Pharmacy, Marmara University, Istanbul 34450, Turkey; dogatopkan@gmail.com; 4 Department of Radiation Oncology, School of Medicine, Koc University, Istanbul 34450, Turkey; ssenyurek@kuh.ku.edu.tr (S.S.); ugurselek@yahoo.com (U.S.); 5 Department of Radiation Oncology, School of Medicine, Afyonkarahisar Health Sciences University, Afyonkarahisar 03030, Turkey; duriyeozturk07@gmail.com

**Keywords:** radiation-induced trismus, heat shock protein 27 (HSP27), head and neck radiotherapy, radiation fibrosis, masticatory muscle injury, oxidative stress, fibro-atrophic remodeling, radiation toxicity

## Abstract

Radiation-induced trismus (RIT) is a common and function-limiting late complication of radiotherapy for head and neck cancers, particularly when the masticatory muscles and temporomandibular joint receive high doses. Despite advances in intensity-modulated radiotherapy, RIT remains a significant survivorship problem, and robust biological biomarkers capable of predicting individual susceptibility are lacking. Heat shock protein 27 (HSP27; HSPB1) is a small heat shock protein that regulates multiple cellular stress responses, including proteostasis, cytoskeletal dynamics, redox homeostasis, apoptosis, and inflammatory signaling. In head and neck malignancies, HSP27 overexpression has been associated with treatment resistance and fibrosis-prone tissue remodeling. Experimental studies further demonstrate that HSP27 promotes transforming growth factor-β-mediated myofibroblast differentiation and extracellular matrix deposition, whereas pharmacologic or genetic inhibition attenuates radiation- or bleomycin-induced pulmonary fibrosis in vivo. Evidence from skeletal muscle biology also indicates that HSP27 modulates muscle integrity, denervation-associated atrophy, inflammatory signaling, and cytoskeletal stability. Although HSP27 has been widely investigated in radiation responses, fibrosis, and skeletal muscle stress adaptation, its potential involvement in the pathogenesis of RIT has not been systematically examined. This review proposes a conceptual framework in which HSP27 functions as an integrative molecular mediator linking radiation-induced oxidative stress, endothelial injury, and fibro-atrophic remodeling within the masticatory apparatus. By integrating current evidence on the epidemiology, dosimetric determinants, imaging correlates, and pathophysiology of RIT with the structural and functional biology of HSP27, this review provides the first tissue-specific synthesis of molecular stress signaling and clinical mechanisms relevant to RIT susceptibility. We further suggest that HSP27 signaling may influence susceptibility to fibro-neuromuscular injury in irradiated masticatory tissues. Given the absence of direct experimental or clinical evidence in this setting, these considerations are derived from mechanistic convergence across related biological systems and should be interpreted as biologically plausible but unproven, with potential implications for future biomarker development and biologically informed prevention strategies.

## 1. Introduction: Radiation-Induced Trismus as an Unresolved Clinical and Biological Challenge

Radiation-induced trismus (RIT) is a clinically significant late effect of radiotherapy or chemoradiotherapy in head and neck cancer (HNC). It is commonly defined as a maximal interincisal opening of ≤35 mm and affects essential functions, including oral intake, speech, and dental care [1]. Contemporary reports indicate that trismus develops in 15–60% of patients, with some individuals continuing to deteriorate more than a decade after treatment [2,3,4,5].

A substantial body of dosimetric research demonstrates that radiation dose to the masticatory apparatus—particularly the medial pterygoid and masseter muscles—is the dominant predictor of RIT. Multiple studies have reported strong dose–volume correlations, with mean doses exceeding approximately 40–50 Gy associated with significantly increased risk [6,7,8,9,10]. Modern IMRT and adaptive radiotherapy approaches have reduced radiation exposure to these muscles; however, trismus still develops in a considerable proportion of patients (8–44%) [11], suggesting that anatomical and dosimetric factors alone cannot fully explain individual susceptibility [12]. Magnetic resonance imaging (MRI)-based evaluations have corroborated these observations by revealing post-treatment volume loss, fatty infiltration, and T2 signal abnormalities consistent with fibrosis and neuromuscular injury [13]. More recently, diffusion-weighted imaging has shown that elevated pretreatment masseter apparent diffusion coefficient values may identify patients at heightened risk before therapy begins [14]. Collectively, these findings indicate that RIT is a multifactorial process involving radiation-induced fibrosis, chronic low-grade inflammation, microvascular injury, and neuromuscular dysfunction throughout the masticatory apparatus [15,16,17].

In parallel, small heat shock proteins (*sHSPs*), particularly heat shock protein 27 (*HSP27*), have attracted increasing attention for their roles in cellular stress adaptation, cytoskeletal stabilization, and tissue resilience. Structural and mechanistic studies show that *HSP27* participates in protein quality control, redox regulation, and cytoskeletal remodeling, while its phosphorylation-dependent oligomerization governs these functions [18,19,20,21]. In cancer biology, *HSP27* has emerged as a regulator of treatment resistance, promoting cell survival under genotoxic, oxidative, and inflammatory stress [22,23]. Studies in head and neck squamous cell carcinoma demonstrate that *HSP27* inhibition enhances radiosensitivity and increases apoptosis following irradiation, whereas forced expression protects tumor cells from radiation-induced damage [24,25,26]. Additional work indicates that *HSP27* modulates pathways involved in epithelial–mesenchymal transition, apoptosis resistance, and NF-κB activation, mechanisms relevant to both tumor biology and radiation-induced tissue remodeling [27,28,29]. Its overexpression in oral squamous cell carcinoma further supports a role in cellular stress adaptation and aggressive tumor behavior [30].

Beyond oncology, *HSP27* also plays important roles in skeletal muscle biology. It mitigates denervation-induced atrophy, suppresses *NF-κB*-driven catabolic signaling, limits oxidative damage, and stabilizes cytoskeletal structures under mechanical strain [31,32,33,34]. These functions are particularly relevant given that RIT involves fibrotic contracture, neuromuscular dysfunction, and impaired muscle regeneration. Although *HSP27* has not been directly studied in irradiated masticatory muscle, evidence from skeletal muscle models suggests that *HSP27* activity may influence how muscle fibers respond to radiation-induced stress. Closely related chaperones such as *HSP7* have also been shown to protect muscle from contraction-induced injury, supporting a broader role of *HSPs* in muscular resilience [35].

Despite extensive research on the pathobiology of radiation-induced trismus (RIT) and the cellular stress-response functions of *HSP27*, the potential intersection between these two fields has not been systematically examined. Existing literature has evaluated *HSP27* primarily in tumor treatment resistance, generalized radiation injury, and fibrosis of non-masticatory tissues, yet no dedicated review has assessed whether these mechanisms may contribute to inter-individual susceptibility to RIT. This review addresses that gap by integrating current clinical evidence on RIT with mechanistic insights from radiation biology, fibrosis, and skeletal muscle stress adaptation. This review therefore proposes a conceptual framework in which *HSP27* may function as an integrative molecular mediator linking oxidative stress, cytoskeletal disruption, endothelial injury, and fibro-atrophic remodeling within the masticatory apparatus. Given these converging observations, *HSP27* represents a biologically plausible—yet still hypothetical—determinant of susceptibility to RIT. Determining whether *HSP27* contributes to fibrosis, neuromuscular injury, or maladaptive repair responses in irradiated masticatory tissues will require targeted mechanistic investigation. Accordingly, this review synthesizes available clinical and mechanistic evidence to explore the potential role of *HSP27* in RIT pathogenesis and to outline priorities for future translational and clinical research. Importantly, direct experimental or clinical evidence linking *HSP27* to RIT is currently lacking. Therefore, the proposed framework is derived from mechanistic convergence across non-RIT biological systems and should be interpreted as hypothesis-generating rather than confirmatory. Accordingly, this review explicitly distinguishes between evidence derived from established experimental and clinical studies and hypothesis-driven inferences specific to RIT; all proposed mechanistic links involving *HSP27* should be considered biologically plausible but unproven.

## 2. Radiation-Induced Trismus: Clinical, Dosimetric, and Pathophysiologic Overview

### 2.1. Epidemiology and Clinical Impact

RIT typically develops within the first 6–12 months following chemoradiotherapy (CRT), although a substantial proportion of patients continue to worsen for many years. Several longitudinal cohort studies have demonstrated the chronicity and clinical burden of this complication. Kraaijenga et al. reported that nearly 38% of long-term survivors had persistent trismus more than a decade after CRT, with significant associations with dysphagia, malnutrition, and impaired quality of life [2]. Systematic reviews confirm wide variability in incidence—ranging from approximately 15% to over 60%—depending on primary site, treatment modality, and follow-up duration [5].

Large comparative analyses demonstrate that multimodal therapy increases the risk of trismus, with CRT regimens involving high doses to the oropharyngeal and oral cavity regions particularly detrimental [31,32]. Beyond functional impairment, RIT interferes with essential survivorship care: it complicates dental surveillance, increases risk of osteoradionecrosis due to limited oral access, and restricts endoscopic and anesthetic procedures [33,34,35].

Additional population-level and trial-based studies strengthen this picture. Kent et al. reported a 45% prevalence of trismus among head and neck cancer patients treated with RT, with clear quality-of-life consequences [11]. Lindblom et al., analyzing long-term outcomes in the ARTSCAN trial, demonstrated persistent trismus several years after treatment, underscoring the chronic nature of this toxicity [36]. A comprehensive meta-analysis by Watters et al. showed a pooled trismus prevalence of 24–47% across RT-treated HNC cohorts, highlighting wide inter-study heterogeneity driven by differences in RT technique, dosimetry, and assessment criteria [37]. Furthermore, contemporary dose-volume studies reaffirm that mean dose to the masseter and pterygoid muscles strongly predicts RIT, as shown by Rao et al. and Kamal et al. [7,9,38].

Collectively, these findings demonstrate that RIT is both common and clinically significant, exerting a sustained impact on oral function, nutrition, and quality of life. Despite advances in radiotherapy technology, RIT remains an important, under-recognized survivorship challenge requiring improved mechanistic understanding, personalized risk prediction, and targeted preventive strategies.

### 2.2. Dose–Volume Relationships of the Masticatory Apparatus

A substantial body of dosimetric evidence demonstrates that the masticatory apparatus—particularly the masseter and medial pterygoid muscles—is central to the development of RIT. Across multiple independent cohorts, these structures display the strongest and most reproducible dose–response associations. Early work using 3D conformal and IMRT techniques found that mean doses exceeding approximately 40–50 Gy to the medial pterygoid and masseter were associated with substantially increased risk of trismus [6,7,8,9,10]. More detailed dose–volume histogram (DVH) analyses have since refined these estimates, confirming that both mean dose and high-dose subvolumes to these muscles are predictive of reduced mandibular mobility following treatment [7,9,10].

Prospective and retrospective studies continue to highlight the medial pterygoid’s vulnerability. This muscle has repeatedly emerged as the single most sensitive structure, with its mean dose showing the strongest correlation with maximal interincisal opening loss and patient-reported symptoms [7,10]. The masseter demonstrates a similar dose–response pattern, with elevated mean dose and larger V60–V70 subvolumes associated with higher RIT prevalence and severity [8,9]. Lateral pterygoid involvement—which plays a key role in mandibular protrusion and deviation—has become increasingly recognized in modern analyses, particularly in series utilizing advanced functional assessments or patient-reported outcomes [7,9].

Studies focusing on advanced radiotherapy techniques also support these patterns. Even in IMRT and VMAT settings—where improved conformality reduces incidental dose to non-target tissues—substantial rates of RIT remain when mean medial pterygoid or masseter doses exceed ~40 Gy [11,12]. Conversely, patients treated with modern approaches who achieve lower doses to these structures demonstrate markedly reduced rates of RIT, reinforcing the importance of individualized contouring and adherence to dose constraints [12]. High-resolution MRI evaluations further corroborate these dosimetric findings by demonstrating post-treatment fatty atrophy, denervation, and fibrotic changes preferentially affecting muscles that received higher radiation exposure [13].

These observations support a consistent mechanistic model: radiation exposure initiates a cascade of microvascular injury, chronic inflammation, and fibro-atrophic remodeling in a dose-dependent fashion, ultimately reducing muscle elasticity and function [16,17,18]. The medial pterygoid and masseter are particularly susceptible due to their high tonic activity, dense Type I fiber composition, and proximity to high-dose target volumes in most head-and-neck cancer radiotherapy plans. When combined with patient-specific biological factors—such as baseline muscle integrity, comorbidities, or molecular susceptibility—these dose–volume effects contribute to the heterogeneous clinical presentation of RIT. However, these dose–volume relationships alone do not fully explain the selective vulnerability of specific masticatory muscles or the observed inter-individual variability in RIT risk.

The preferential susceptibility of the masseter and pterygoid muscles to RIT likely reflects a convergence of anatomical exposure, biomechanical demand, and intrinsic stress-response biology that extends beyond dosimetric considerations alone [7,8,9,10]. These muscles are consistently encompassed within, or immediately adjacent to, high-dose regions in head and neck radiotherapy—particularly in nasopharyngeal and oropharyngeal cancers—resulting in reproducible exposure to intermediate-to-high radiation doses across techniques [7,9,10]. However, the observation that RIT develops in only a subset of patients with comparable dose distributions suggests that tissue-specific biological properties modulate vulnerability within the masticatory apparatus.

From a physiological perspective, the masseter and pterygoid muscles possess distinctive functional and metabolic characteristics, including sustained tonic activity, repetitive high-load contractions during mastication, and a relatively high proportion of oxidative (Type I) muscle fibers, characterized by elevated mitochondrial density and oxidative metabolism [39]. These features are associated with continuous generation of reactive oxygen species (ROS) and a reliance on tightly regulated redox homeostasis and cytoskeletal integrity to maintain contractile function. In skeletal muscle, *HSP27* is preferentially expressed and dynamically phosphorylated in response to mechanical strain and metabolic stress, where it stabilizes actin filaments and protects myofibrillar structure [34], whereas reduced *HSP27* activity has been associated with increased oxidative injury and accelerated muscle dysfunction [40,41].

In the setting of radiotherapy, these intrinsic properties may create a biologically permissive environment for the amplified and sustained activation of *HSP27*. Ionizing radiation induces *ROS* accumulation, endothelial injury, and inflammatory signaling, which converge on the *p38 MAPK–MAPKAPK2/3* pathway to drive *HSP27* phosphorylation [42,43,44,45]. In muscles already characterized by high baseline oxidative flux and continuous mechanical loading, such as the masseter and pterygoids, this additional stress burden may exceed adaptive thresholds, leading to prolonged or dysregulated *HSP27* activity. While acute *HSP27* activation is likely cytoprotective—preserving cytoskeletal integrity and cellular viability—persistent activation within a chronically irradiated microenvironment may promote survival of structurally compromised fibroblasts and myocytes, facilitate cytoskeletal rigidity, and sustain profibrotic signaling through pathways including *TGF*-*β* and *NF-κB* [17,29,46,47,48,49,50].

These effects are further compounded by the unique microenvironment of irradiated masticatory muscles, where microvascular rarefaction, chronic hypoxia, satellite cell depletion, and neuromuscular junction disruption collectively impair regenerative capacity [16,17]. Under such conditions, the balance between adaptive repair and maladaptive remodeling may shift toward a fibro-atrophic phenotype characterized by extracellular matrix accumulation, reduced muscle elasticity, and progressive contracture. Importantly, *HSP27*-mediated inhibition of apoptosis and stabilization of cytoskeletal elements—beneficial in the acute phase—may, in the chronic setting, permit persistence of dysfunctional cellular populations and reinforce structural stiffness.

Collectively, these observations support a tissue-specific vulnerability model in which the masseter and pterygoid muscles serve as a convergence point for high radiation exposure, continuous mechanical demand, oxidative metabolic stress, and HSP27-dependent stress-response signaling. This integrated framework suggests that susceptibility to RIT is not uniformly distributed across skeletal muscle groups but instead reflects a context-dependent interaction between radiation-induced injury and intrinsic muscle biology. Within this paradigm, *HSP27* may function as a context-sensitive amplifier of fibro-neuromuscular remodeling in the masticatory apparatus, providing a biologically plausible—yet currently unproven—mechanistic basis for the selective involvement of these muscles in RIT. The structural and functional consequences of these processes are further elucidated by advanced imaging modalities, which provide insight into the evolution of fibro-neuromuscular injury in irradiated masticatory tissues.

### 2.3. Imaging Correlates and Structural Pathology

Cross-sectional imaging—particularly magnetic resonance imaging (MRI)—has become central to characterizing structural and functional deterioration within the masticatory apparatus following radiotherapy. Across nasopharyngeal carcinoma (NPC) and other head and neck cancer treatment settings, MRI consistently reveals a constellation of post-treatment alterations, including muscle volume loss, increased T2 signal intensity, fatty infiltration, and distortion of normal fascial planes—features that collectively reflect fibrotic remodeling, chronic inflammation, and denervation-associated atrophy [13,18]. Arpacı et al. demonstrated pronounced volume loss in both the masseter and pterygoid muscles in NPC patients, with the lateral pterygoid frequently exhibiting marked T2 hyperintensity suggestive of chronic edema and neuromuscular compromise [13].

Diffusion-weighted imaging (DWI) has emerged as a particularly sensitive modality for detecting early microstructural abnormalities. In a prospective cohort, Pehlivan et al. showed that elevated pretreatment masseter apparent diffusion coefficient (ADC) values independently predicted later development of RIT, implying that subtle subclinical architectural or vascular vulnerabilities may predate overt fibrosis [14]. Subsequent DWI-based analyses have provided complementary insights, demonstrating that persistently increased ADC values correlate with long-term reductions in mandibular mobility and imaging patterns consistent with progression from early edematous injury to late fibrotic contracture [34,39].

Functional imaging further strengthens this structural framework. Johansson et al. demonstrated that altered masseter electromyography (EMG) profiles following radiotherapy closely parallel MRI-defined tissue remodeling, linking radiologic abnormalities directly to neuromuscular dysfunction and reduced mandibular mobility [18]. Dynamic MRI and cine-sequence studies similarly reveal reduced excursion, asymmetric muscle activation, and delayed relaxation kinetics—functional consequences that align closely with the fibro-atrophic cascade described in radiation injury models [16,17].

Advanced imaging techniques have further clarified the mechanistic interpretation of these findings. High-resolution morphological MRI demonstrates early expansion of extracellular matrix compartments and disruption of normal pennate fiber orientation within months after radiotherapy [51]. Quantitative T2-mapping studies show strong correlations between elevated T2 values and chronic myofiber loss with fatty replacement during later phases of injury [52]. CT-based radiomics analyses have identified textural heterogeneity—particularly entropy and gray-level nonuniformity—as predictors of subsequent trismus, reflecting subclinical fibrosis and microvascular rarefaction [53]. PET/MRI investigations report increased FDG uptake in chronically irradiated masticatory muscles, supporting the presence of persistent metabolic activity and low-grade inflammation in sustaining progressive contracture [54]. Additional advanced MRI approaches, including elastography and diffusion tensor imaging, similarly demonstrate progressive increases in tissue stiffness and reductions in fiber directional integrity over time [13,55,56,57].

Although direct histopathologic correlation in RIT remains limited, imaging findings across these studies converge on a consistent pathophysiologic pattern characterized by collagen accumulation, persistent myofibroblast activity, microvascular rarefaction, and progressive denervation-related atrophy within irradiated masticatory tissues [16,17]. These radiologic correlates underscore that RIT is not merely a mechanical limitation of mandibular excursion but rather a biologically active process involving chronic connective-tissue remodeling and neuromuscular degeneration.

Collectively, the imaging evidence supports a unified pathophysiologic model in which radiation initiates microvascular injury, inflammatory activation, and myofiber degeneration, ultimately culminating in fibrotic replacement of contractile tissue. Radiologically, these changes manifest as T2 hyperintensity, diffusion abnormalities, atrophy, and fatty infiltration, while functionally they produce impaired mandibular excursion and reduced muscle recruitment. The convergence of structural, functional, metabolic, and biomechanical imaging, therefore, reinforces that RIT represents a sustained fibro-neuromuscular disorder shaped by both radiation exposure and individual biological susceptibility. Understanding the biological mechanisms underlying these structural and functional alterations is essential for clarifying the pathophysiology of RIT and for guiding subsequent mechanistic inquiry.

### 2.4. Pathophysiologic Framework

RIT develops through a complex interplay of biological processes in which fibrosis, skeletal muscle degeneration, neuromuscular dysfunction, inflammation, and microvascular compromise evolve concurrently rather than in a strictly linear sequence. Although radiation dose to the masticatory apparatus remains the principal determinant of risk, substantial clinical variability among patients receiving similar dosimetric exposures suggests that intrinsic biological susceptibility contributes meaningfully to disease expression. A central component of this susceptibility is the chronic radiation-induced fibro-atrophic response, characterized by persistent activation of transforming growth factor-β (*TGF-β*), fibroblast proliferation, and excessive collagen deposition that progressively stiffens the masseter, pterygoid muscles, and periarticular connective tissues. Experimental studies of radiation fibrosis further demonstrate that early endothelial injury, microvascular rarefaction, oxidative stress, and chronic low-grade inflammation act as upstream triggers of sustained fibroblast activation and myofibroblast persistence, ultimately replacing contractile tissue with dense extracellular matrix and reducing mandibular mobility [16,17].

Concurrently, skeletal muscle remodeling amplifies functional decline. Radiation induces myofiber injury, mitochondrial dysfunction, satellite cell depletion, and impaired regenerative capacity, promoting atrophy, fatty infiltration, and reduced contractile efficiency. Progressive hypoxia—driven by endothelial loss and capillary rarefaction—further disrupts oxidative metabolism and myogenic repair, while denervation and neuromuscular junction degradation contribute additional functional deficits.

These neuromuscular alterations, demonstrated in functional electromyographic studies [18], parallel the structural abnormalities observed in MRI-based investigations and indicate that RIT represents a biologically active fibro-neuromuscular syndrome rather than a purely mechanical reduction in mouth opening. The convergence of fibrotic, vascular, and neuromuscular processes provides a mechanistic basis for the progressive, heterogeneous clinical course observed in patients.

Within this interconnected system, fibroblast growth factor-2 (*FGF-2*) has emerged as a potential modulator of tissue remodeling. Under physiological conditions, *FGF-2* supports angiogenesis, satellite-cell activation, and coordinated tissue repair; however, in irradiated tissues, dysregulated or prolonged *FGF-2* activity has been associated with aberrant fibroblast proliferation, extracellular matrix expansion, and impaired regenerative signaling in experimental models [58,59,60,61]. In the masticatory apparatus—where radiation induces simultaneous hypoxia, inflammation, and neuromuscular injury—maladaptive *FGF-2* signaling may further shift the balance toward fibrosis and contracture, thereby contributing to RIT development.

Systemic inflammatory and nutritional states also influence susceptibility to trismus. Emerging clinical evidence suggests that inflammatory burden and nutritional reserve modify tissue responses to radiotherapy. Elevated Global Immune-Nutrition-Inflammation Index values [62], adverse pan-immune-inflammatory profiles [63], and reduced hemoglobin levels reflecting impaired oxygen-carrying capacity [64,65] have each been associated with increased RIT risk.

These observations support the concept that RIT arises from an interaction between local radiation-induced tissue injury and systemic biological factors, rather than from a purely locoregional toxicity. Variability in inflammatory burden, oxygen delivery, and metabolic reserve may therefore influence both the initiation and progression of fibro-neuromuscular remodeling.

Microvascular injury represents a final unifying mechanism. Endothelial cell loss, impaired perfusion, and chronic hypoxia perpetuate oxidative stress and inflammatory signaling, sustaining fibroblast activation and extracellular matrix deposition. These vascular abnormalities correspond closely with advanced imaging findings demonstrating progressive fibrosis, fatty infiltration, and denervation-related atrophy in irradiated masticatory muscles [13,51,52,53,54,55,56,57]. Importantly, the temporal progression documented in longitudinal MRI cohorts parallels the gradual clinical deterioration observed in many patients.

Collectively, these interconnected biological processes—fibrosis, hypoxia, inflammation, neuromuscular degeneration, and growth-factor dysregulation—create a pathophysiologic environment in which cellular stress-response systems may act as critical modulators of tissue fate after irradiation. Among candidate mediators capable of integrating these pathways, *HSP27* is particularly noteworthy. *HSP27* regulates redox homeostasis, cytoskeletal stability, apoptosis resistance, inflammatory signaling, and fibroblast/myofibroblast activity—functions that closely overlap with the mechanisms implicated in RIT development. Moreover, *HSP27* modulates cellular survival under conditions characteristic of irradiated muscle, including oxidative injury, cytokine activation, hypoxia, and mechanical strain.

**Figure 1:**
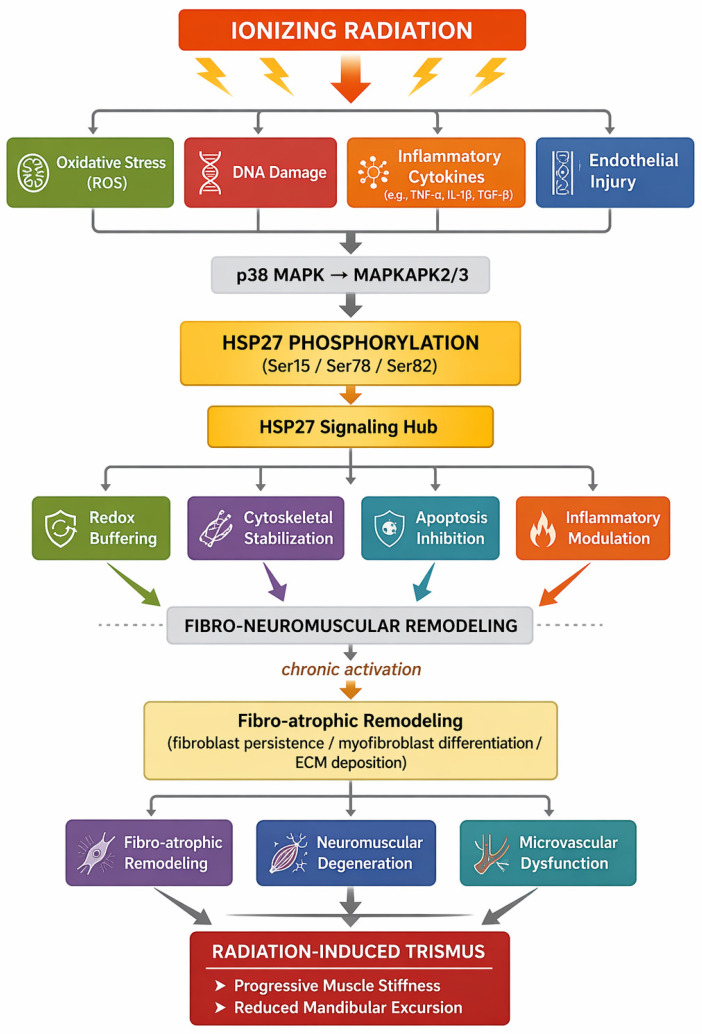
Proposed mechanistic cascade linking radiotherapy-induced cellular stress to radiation-induced trismus (RIT). Ionizing radiation activates oxidative stress, endothelial injury, inflammatory signaling, and the *p38 MAPK–MAPKAPK2/3* pathway, leading to *HSP27* phosphorylation. Persistent activation may promote fibro-neuromuscular remodeling, muscle stiffness, and reduced mandibular excursion. This figure integrates evidence from non-RIT systems and represents a hypothesis-generating framework. Created with BioRender.com. https://BioRender.com/ab8i4n5 (accessed on 23 March 2026). Note: Ionizing radiation elicits a spectrum of cellular stress responses within the masticatory apparatus, including oxidative stress, DNA damage, inflammatory cytokine signaling, and endothelial injury. These stressors converge to activate the *p38 MAPK–MAPKAPK2/3* signaling pathway, resulting in phosphorylation and functional activation of heat shock protein 27 (*HSP27*). In experimental systems, phosphorylated HSP27 functions as a dynamic stress-response regulator that modulates redox homeostasis, cytoskeletal stability, resistance to apoptosis, and inflammatory signaling. Under conditions of persistent injury, microvascular compromise, and impaired regenerative capacity, sustained *HSP27* activation may contribute to fibro-neuromuscular remodeling characterized by myofibroblast persistence, extracellular matrix accumulation, neuromuscular degeneration, and microvascular dysfunction. The convergence of these processes is hypothesized to culminate in progressive muscle stiffness, restricted mandibular excursion, and the clinical phenotype of radiation-induced trismus (RIT). This schematic represents a hypothesis-generating, tissue-contextualized mechanistic framework derived from convergent evidence across non-RIT biological systems and should therefore be interpreted as biologically plausible but unproven.

Within this context, the molecular and physiological architecture underlying RIT provides a rationale for examining *HSP27* as a potential biomarker, mediator, or amplifier of radiation-induced late toxicity. The subsequent sections, therefore, explore how *HSP27* biology intersects with the fibro-neuromuscular pathways outlined above and whether modulation of *HSP27* may inform future strategies for risk stratification and therapeutic intervention in RIT (Figure 1).

Given the absence of RIT-specific molecular studies, the following sections synthesize indirect evidence from radiation biology, fibrosis, and skeletal muscle research to construct a biologically plausible—yet unproven—mechanistic framework. Rather than establishing causality, this approach aims to identify mechanistic convergence points through which *HSP27* may influence radiation-induced fibro-neuromuscular injury. These inferences are therefore hypothesis-generating and should be interpreted with caution, pending validation in tissue-specific experimental models and clinical investigations.

A stepwise interpretation of this proposed framework may be outlined as follows (Figure 1): (1) ionizing radiation induces oxidative stress, DNA damage, endothelial injury, and inflammatory cytokine signaling within the masticatory apparatus; (2) these stressors activate the *p38 MAPK–MAPKAPK2/3* signaling cascade, resulting in phosphorylation and functional activation of *HSP27*; (3) activated *HSP27* enhances cytoskeletal stabilization, redox buffering, and apoptosis resistance in irradiated fibroblasts, endothelial cells, and myocytes; (4) under conditions of persistent injury, hypoxia, and impaired regeneration, sustained *HSP27* activation may promote fibroblast persistence and myofibroblast differentiation while amplifying profibrotic signaling pathways, including *TGF-β* and *NF-κB* [17,29,61,62,63,64,65,66]; (5) these processes drive extracellular matrix accumulation, microvascular dysfunction, and neuromuscular degeneration within the masseter and pterygoid muscles; and (6) the cumulative effect is progressive muscle stiffening, reduced mandibular excursion, and the clinical phenotype of RIT. This sequence represents a hypothesis-generating mechanistic model derived from convergent evidence across non-RIT systems and should be interpreted as biologically plausible but unproven.

## 3. Biology of Heat Shock Protein 27

### 3.1. Structural Properties and Regulation

*HSP27*, also known as *HSPB1*, is a highly conserved member of the *sHSP* family and is structurally defined by a central α-crystallin domain flanked by intrinsically disordered N- and C-terminal regions. These terminal segments confer remarkable structural dynamism, enabling rapid transitions between large oligomeric assemblies and smaller, more active chaperone units. Oligomer size and chaperone activity are governed by phosphorylation-dependent rearrangements within the *HSP27* complex, allowing the protein to function as a rapid sensor and integrator of cellular stress.

Phosphorylation at key serine residues—*Ser15*, *Ser78*, and *Ser82*—is mediated primarily through the p38 MAPK–MAPKAPK2/3 signaling cascade, a canonical stress-response pathway activated by oxidative stress, inflammatory cytokines, heat shock, and ionizing radiation [66,67]. These phosphorylation events destabilize large oligomers and promote the formation of smaller, more mobile species with altered client-binding affinity. Structural and biophysical studies demonstrate that this phosphorylation-driven remodeling regulates intersubunit interactions within *HSP27*, thereby modulating oligomeric equilibrium and substrate recognition [68,69,70].

Beyond its classical chaperone activity, *HSP27* plays a central role in regulating cytoskeletal architecture. Phosphorylated *HSP27* interacts with actin filaments and cytoskeletal regulators, influencing actin polymerization dynamics and stabilizing filamentous actin under stress conditions. Pioneering studies by Landry and Huot demonstrated that *HSP27*-mediated modulation of actin assembly preserves cellular morphology and motility during oxidative or mechanical injury [43]. These cytoskeletal regulatory functions are particularly relevant in tissues subjected to repetitive mechanical loading, such as skeletal muscle and the masticatory apparatus.

*HSP27* also participates in regulating cell survival pathways. Mechanistic studies demonstrate that *HSP27* inhibits apoptosis by binding cytochrome c, preventing apoptosome assembly, and suppressing procaspase-3 activation [71]. Additional evidence indicates that *HSP27* interferes with *DAXX*-, *Fas*-, and mitochondrial stress-mediated apoptotic signaling pathways [72]. These anti-apoptotic functions become markedly enhanced during radiation exposure, when rapid phosphorylation of *HSP27* augments cellular survival under genotoxic stress.

Together, these structural and regulatory characteristics position *HSP27* as a highly adaptable stress-response protein capable of integrating proteotoxic stress, cytoskeletal remodeling, oxidative signaling, and the control of apoptosis. These properties explain its well-established role in treatment resistance in cancer and provide a conceptual framework for exploring how *HSP27* might influence susceptibility to radiation-induced tissue injury. Such phosphorylation-dependent and cytoskeletal regulatory functions may be particularly relevant in irradiated contractile tissues exposed to chronic oxidative and mechanical stress, including the masticatory apparatus affected in radiation-induced trismus.

### 3.2. Canonical Cellular Functions

*HSP27* exerts a broad repertoire of cellular functions, positioning it as a central mediator of stress resilience in both malignant and normal tissues. As a molecular chaperone, it stabilizes unfolded or partially denatured proteins, prevents irreversible aggregation, and cooperates functionally with *HSP70/HSP90* networks to maintain proteostasis during oxidative, metabolic, or genotoxic stress [66,69,70]. This versatility arises from its phosphorylation-dependent oligomeric plasticity: activation of the *p38 MAPK* pathway reshapes *HSP27* oligomer size and client-binding capacity, enabling rapid adaptation to fluctuating cellular stress conditions [66,67,68].

A defining feature of *HSP27* biology is its regulation of actin cytoskeletal dynamics. Through direct interaction with actin monomers and filaments, non-phosphorylated *HSP27* stabilizes actin structures and limits polymerization, whereas phosphorylation promotes filament turnover and cytoskeletal remodeling [43]. These activities preserve cellular structural integrity under mechanical deformation, oxidative stress, or radiation-induced cytoskeletal injury—conditions particularly relevant in contractile tissues such as skeletal muscle.

*HSP27* also plays a major role in redox homeostasis. Maintaining glutathione balance and stabilizing redox-sensitive proteins limits intracellular reactive oxygen species (*ROS*) accumulation and protects mitochondria from oxidative injury [71]. Through these mechanisms, *HSP27* indirectly modulates downstream signaling pathways that regulate inflammation and fibrosis, including *NF-κB-* and *TGF-β*-mediated transcriptional programs.

In addition, *HSP27* exerts potent anti-apoptotic control. It inhibits cytochrome-c-dependent caspase-3 activation, interferes with apoptosome assembly, and modulates procaspase-9 and *DAXX* signaling pathways [71,72]. These actions enhance the survival of stressed fibroblasts, endothelial cells, epithelial cells, and myocytes—cell populations that participate in the long-term remodeling responses following radiation exposure.

*HSP27* further interfaces with regulatory pathways implicated in chronic tissue injury and fibrosis, including *NF-κB*-mediated cytokine signaling and *TGF-β*-driven myofibroblast activation [66,67]. Through these interactions, it influences extracellular matrix turnover, fibroblast persistence, and inflammatory signaling—processes central to radiation-induced fibro-atrophy.

Taken together, these integrated biochemical properties position *HSP27* as a potential upstream modulator of several biological processes implicated in RIT pathogenesis. Its regulation of cytoskeletal integrity may influence how irradiated masticatory muscles withstand chronic mechanical loading and microvascular compromise, while its antioxidant and anti-apoptotic activities may permit survival of dysfunctional fibroblasts and myocytes that contribute to progressive fibrosis. By engaging oxidative stress, *NF-κB*, and *TGF-β* signaling pathways—key drivers of the fibro-atrophic cascade described in Section 2 [16,17]—*HSP27* may act as an integrative regulator linking radiation-induced cellular stress to extracellular matrix accumulation and neuromuscular degeneration. In the context of RIT, such functions may influence the persistence of injured fibroblasts, endothelial cells, or myocytes, thereby modulating the balance between adaptive repair and chronic fibro-neuromuscular remodeling. These mechanistic intersections provide a coherent biological foundation for hypothesizing that inter-individual differences in *HSP27* expression or activation could influence the onset, severity, and chronic progression of RIT.

### 3.3. HSP27 Expression Patterns in Normal Tissues and Muscle

HSP27 is constitutively expressed across a broad range of human tissues, with particularly high abundance in skeletal muscle, cardiac muscle, and epithelial cell layers, where it functions as an early protective mediator against mechanical deformation, oxidative stress, and metabolic injury [34,35,46,70]. Within skeletal muscle, *HSP27* expression is not uniform across fiber types. Type I (slow-twitch, oxidative) fibers—which sustain prolonged contractile activity and continuous mitochondrial ROS generation—exhibit higher baseline *HSP27* expression than fast-twitch glycolytic fibers [73,74]. This distribution suggests an important role for *HSP27* in maintaining cytoskeletal stability and redox balance under conditions of sustained mechanical and metabolic demand.

*HSP27* expression is also dynamically regulated by mechanical loading and contractile activity. Exercise, eccentric loading, and stretch-induced microtrauma rapidly induce *HSP27* phosphorylation in both experimental models and human muscle, promoting actin remodeling, sarcomeric stabilization, and increased cytoskeletal compliance [34,73]. Conversely, during disuse, immobilization, or denervation, skeletal muscle initially upregulates *HSP27* as part of an early stress-response program designed to buffer cytoskeletal instability. However, prolonged denervation ultimately leads to impaired *HSP27* induction, reduced antioxidant capacity, and accelerated myofiber degeneration [74,75].

Age-related alterations further influence *HSP27* biology. In aging skeletal muscle, both baseline expression and phosphorylation responsiveness of *HSP27* decline, contributing to diminished proteostatic capacity, increased oxidative stress burden, and heightened susceptibility to contraction-induced injury [76]. These changes parallel vulnerabilities observed in irradiated tissues, where impaired regenerative capacity and compromised redox homeostasis similarly accelerate structural deterioration.

These physiological features are directly relevant to the masticatory apparatus. The masseter and pterygoid muscles are highly active skeletal muscles that sustain repetitive mechanical loading during mastication and speech, requiring continuous cytoskeletal stabilization and management of oxidative stress. Radiation exposure disrupts this balance by inducing chronic oxidative stress, microvascular compromise, neuromuscular junction degeneration, and progressive extracellular matrix remodeling. Under such conditions, *HSP27* would normally be expected to function as a protective stress-response protein.

Variability in baseline *HSP27* expression, phosphorylation capacity, or inducibility may therefore influence how masticatory muscles respond to irradiation. Individuals with robust *HSP27*-mediated stress responses may maintain cytoskeletal integrity and adaptive remodeling, whereas impaired *HSP27* signaling could permit progressive fibrosis, neuromuscular dysfunction, and structural degeneration. Such variability provides a biologically plausible mechanism through which *HSP27* activity might modulate susceptibility to RIT. Because the masseter and pterygoid muscles are continuously active skeletal muscles, inter-individual differences in *HSP27* expression or inducibility may be particularly relevant to susceptibility to radiation-related injury in the masticatory apparatus. Evidence from experimental studies of radiation injury and fibrotic remodeling further supports this possibility and provides important mechanistic insights into how *HSP27* signaling may influence tissue responses to irradiation.

## 4. *HSP27* in Radiation Responses and Fibro-Neuromuscular Tissue Remodeling: Inference-Based Mechanistic Insights

### 4.1. Induction and Regulation of HSP27 in Irradiated Normal Tissues

Ionizing radiation triggers a rapid, multifaceted stress response in normal tissues, and *HSP27* activation has been reported as one of its earliest and most conserved components across experimental models. Within minutes of exposure, radiation-induced oxidative stress, DNA damage, and membrane perturbation activate upstream kinases in the *p38 MAPK–MAPKAPK2/3* pathway, leading to phosphorylation of *HSP27* at key serine residues (*Ser15*, *Ser78*, *Ser82*) [66,67]. This phosphorylation has been shown to drive a shift from large oligomeric complexes toward smaller, more dynamic assemblies that modulate cytoskeletal stability, stress-fiber remodeling, and chaperone activity in experimental systems [43,68,69]. Through these structural transitions, *HSP27* is thought to become functionally repositioned to stabilize damaged proteins, buffer redox imbalance, and support cytoskeletal integrity in mechanically active tissues.

These observations, however, are derived from non-RIT biological systems, including fibrosis models, skeletal muscle studies, and radiation response research in other tissues. Accordingly, they suggest a role for *HSP27* in early cellular adaptation to radiation-induced stress, but this should be interpreted as inferential rather than directly evidentiary in the context of RIT.

Radiation also alters intracellular redox equilibrium, producing sustained reactive oxygen species (*ROS*) generation that persists long after exposure due to mitochondrial dysfunction and chronic vascular injury. In experimental systems, *HSP27* expression has been shown to increase as part of the antioxidant defense response, contributing to glutathione homeostasis and protecting actin and mitochondrial proteins from oxidative stress [71]. In skeletal muscle—already prone to contraction-induced *ROS* production—this response has been documented in both human and animal models following mechanical load, denervation, or oxidative stress [34,35,46,70,73].

These findings support the biological plausibility that irradiated masticatory muscles may upregulate *HSP27* as a compensatory mechanism to preserve structural and metabolic integrity under chronic oxidative and hypoxic stress; however, this adaptive response has not been directly demonstrated in RIT.

Importantly, radiation injury simultaneously activates inflammatory and profibrotic pathways, including *NF-κB* and *TGF-β* signaling, which intersect with *HSP27* regulatory networks. In multiple experimental systems, *HSP27* has been shown to modulate *NF-κB* activation and influence downstream cytokine responses [66,67], and to regulate cytoskeletal structures that determine fibroblast contractility and myofibroblast differentiation.

Accordingly, *HSP27* may occupy a convergence point at which oxidative stress, inflammatory activation, and cytoskeletal remodeling jointly influence the balance between tissue repair and fibrosis; however, its specific role in irradiated masticatory tissues remains uncharacterized.

In tissues undergoing denervation or impaired neuromuscular signaling—as documented in skeletal muscle after radiotherapy—*HSP27* expression has been observed to increase as part of a protective response to structural disuse, altered contractile loading, and elevated *ROS* production [34,35,76]. This regulatory pattern has been described in experimental and physiological muscle models subjected to denervation and mechanical stress.

Extrapolating from these findings, irradiated masticatory muscles may undergo repeated stress cycles that sustain *HSP27* activation; however, direct evidence supporting this mechanism in RIT is currently lacking.

Taken together, these observations indicate that *HSP27* acts as a dynamically regulated component of cellular stress-response networks under irradiation in a range of biological systems. By integrating redox control, cytoskeletal maintenance, protein quality management, and inflammatory modulation, *HSP27* may influence how irradiated tissues respond to ongoing mechanical load, microvascular insufficiency, and fibro-inflammatory stress.

However, it must be emphasized that these insights are derived from non-RIT contexts, and the role of *HSP27* in RIT remains to be defined. Accordingly, its proposed involvement should be interpreted as biologically plausible but unproven, pending validation in RIT-specific experimental and clinical settings.

Beyond its immediate stress-response functions, the biological effects of *HSP27* in irradiated tissues appear to evolve over time, reflecting a transition from adaptive cytoprotection to potential involvement in chronic tissue remodeling. In the acute phase following radiation exposure, *HSP27* activation is predominantly protective, contributing to protein stabilization, preservation of cytoskeletal integrity, maintenance of redox balance, and inhibition of apoptosis in stressed cells. These functions are consistent with its role as an early responder to oxidative and genotoxic stress, supporting short-term cellular survival.

However, in the setting of persistent radiation-induced injury—characterized by chronic oxidative stress, microvascular compromise, and sustained inflammatory signaling—prolonged *HSP27* activation may exert qualitatively different effects. Experimental evidence from non-RIT systems suggests that sustained *HSP27* signaling may promote fibroblast survival, facilitate myofibroblast differentiation, and stabilize cytoskeletal structures that favor extracellular matrix accumulation and tissue stiffening [46,47,48,49,50]. In this context, mechanisms that are initially protective may, over time, contribute to maladaptive fibro-atrophic remodeling by permitting the persistence of dysfunctional cellular populations and reinforcing profibrotic signaling pathways, including *TGF-β* and *NF-κB*.

Viewed in this light, *HSP27* may be conceptualized as a context- and time-dependent regulator of tissue responses to radiation, in which the balance between protective and pathogenic effects is shaped by both the duration of activation and the surrounding microenvironment [17,29]. In the acute phase, *HSP27* activation may support cellular survival through preservation of proteostasis, cytoskeletal integrity, and redox balance, whereas under conditions of persistent oxidative stress, microvascular compromise, and chronic inflammatory signaling, sustained activation may instead favor fibroblast persistence, maladaptive repair, and progressive extracellular matrix accumulation. This temporal framework therefore provides a coherent basis for understanding how an initially adaptive stress-response mechanism could transition into a contributor to chronic fibro-atrophic remodeling, as discussed in the following section. While direct RIT-specific evidence remains unavailable, these observations provide biologically plausible mechanistic parallels relevant to irradiated masticatory tissues. Reported effects of *HSP27* after irradiation are not uniform across tissues or experimental models, and may vary according to radiation dose, timing, cell type, and local microenvironment.

### 4.2. HSP27 as an Active Modulator of Fibro-Atrophic Remodeling in Irradiated Masticatory Tissues

Across multiple fibrotic disorders, *HSP27* has been reported to function not merely as a passive stress marker but as a regulator of fibroblast phenotype, extracellular matrix dynamics, and tissue stiffening. In irradiated normal tissues, the fibro-atrophic process is initiated by oxidative stress, cytoskeletal disruption, endothelial injury, and inflammatory activation—conditions that have been shown to induce *HSP27* phosphorylation and redistribution to actin-rich cellular compartments. Through this phosphorylation-dependent transition, *HSP27* has been shown in experimental systems to modulate actin turnover and stabilize cytoskeletal structures under oxidative or mechanical strain, thereby influencing how fibroblasts, endothelial cells, and myocytes respond to injury [66,67,68]. These observations, however, are derived from non-RIT biological contexts, including fibrosis models and skeletal muscle studies, and should therefore be interpreted as inferential rather than directly evidentiary in irradiated masticatory tissues.

Evidence from experimental models of fibrosis further supports this regulatory role. Although several fibrosis models implicate *HSP27* in myofibroblast activation, other studies suggest stress proteins may also limit tissue injury or support adaptive repair, indicating that *HSP27*-related responses are not uniformly profibrotic. In pulmonary fibrosis, *HSP27* expression has been shown to increase in activated fibroblasts and to promote transforming growth factor-β (*TGF-β*)-driven myofibroblast differentiation and extracellular matrix deposition [46]. Similar mechanisms have been described in cardiac fibrosis, where *HSP27* interacts with actin-remodeling proteins to sustain myofibroblast persistence and collagen accumulation [47]. Mechanistic studies also demonstrate that phosphorylated *HSP27* protects actin filaments from oxidative damage, promotes stress-fiber formation, and facilitates acquisition of the contractile phenotype characteristic of myofibroblasts [48,49].

These processes share important similarities with the progressive stiffening and contracture observed in masticatory muscles affected by RIT; however, direct evidence supporting such mechanisms in irradiated masticatory tissues is currently lacking.

Radiation further amplifies profibrotic processes by generating chronic reactive oxygen species, inducing microvascular injury, and sustaining low-grade inflammatory signaling. Each of these factors has been associated with increased *HSP27* phosphorylation and expression in experimental systems. In endothelial cells, *HSP27* has been shown to stabilize the cytoskeleton during oxidative stress, limit apoptosis, and promote survival of structurally compromised endothelial populations [20].

While this response may initially preserve vascular integrity, persistent endothelial survival without functional recovery has been proposed to contribute to chronic ischemia and fibroblast activation—features consistent with microvascular rarefaction and hypoxia observed in RIT. However, the role of *HSP27* in mediating these processes within irradiated masticatory tissues remains uncharacterized.

In skeletal muscle, *HSP27* expression has been shown to be induced by contraction-induced injury, denervation, and aging-associated oxidative stress in experimental and physiological models [34,35,75,76]. These conditions share important biological features with irradiated muscle, including mitochondrial dysfunction, cytoskeletal fragility, and impaired regenerative capacity.

Although elevated *HSP27* expression may initially protect damaged myocytes from apoptosis, prolonged survival of dysfunctional muscle fibers has been hypothesized to contribute to structural disorganization and extracellular matrix accumulation. Such changes are consistent with imaging findings in RIT, in which magnetic resonance imaging frequently demonstrates fibrosis, fatty infiltration, and abnormal T2 signal within irradiated masticatory muscles [13,51,52,53,54,55,56,57]; however, a direct mechanistic link to *HSP27* has not been established.

Collectively, these observations suggest that *HSP27* may occupy a relevant position at the intersection of several biological processes implicated in the fibro-atrophic cascade, including oxidative stress regulation, cytoskeletal stabilization, inflammatory signaling, endothelial survival, fibroblast activation, and impaired skeletal muscle regeneration. Importantly, despite these pathways’ involvement in radiation-induced tissue injury, the role of *HSP27* in mediating fibro-atrophic remodeling within irradiated masticatory muscles has not yet been systematically investigated. Accordingly, its proposed contribution should be interpreted as biologically plausible but unproven.

Through these converging mechanisms, *HSP27* may act as a potential upstream modulator of radiation-induced fibrosis and contracture within the masticatory apparatus. By influencing how muscular, endothelial, and stromal cells respond to persistent oxidative and mechanical stress, *HSP27* may affect both susceptibility to and progression of RIT. However, these interpretations remain hypothesis-generating and require validation in tissue-specific experimental models and clinical studies. This conceptual framework, therefore, provides a rationale for evaluating *HSP27* as a candidate biomarker or therapeutic target in radiation-induced fibro-neuromuscular remodeling.

Collectively, these observations indicate that radiation-induced trismus arises from the interaction of dose-dependent tissue injury with inter-individual differences in fibrosis, vascular resilience, neuromuscular repair capacity, and chronic stress signaling. These unresolved biological heterogeneities provide the rationale for evaluating molecular stress mediators such as *HSP27* beyond dosimetry alone. Beyond fibro-atrophic remodeling, radiation injury within the masticatory apparatus also involves progressive neuromuscular degeneration, microvascular compromise, and cytoskeletal destabilization—biological domains in which *HSP27* has been implicated in other systems and which are examined in the following section.

### 4.3. HSP27 in Neuromuscular Degeneration, Microvascular Injury, and Cytoskeletal Failure

RIT arises from the convergence of fibro-atrophic remodeling, neuromuscular degeneration, microvascular compromise, and persistent oxidative–inflammatory stress. As outlined in the preceding sections, these processes evolve in parallel rather than as isolated events, collectively producing progressive deterioration of masticatory muscle structure and function. Within this complex pathobiological environment, *HSP27* has been implicated in multiple cellular stress-response pathways—including cytoskeletal regulation, apoptosis, redox homeostasis, inflammatory signaling, and fibrotic remodeling—in a range of experimental systems. This positioning suggests that *HSP27* may function as an integrative molecular node; however, its role in determining inter-individual susceptibility to RIT remains uncharacterized.

Several key features of *HSP27* biology intersect with mechanisms implicated in radiation-induced fibro-atrophy. Its phosphorylation-dependent regulation of actin dynamics and cytoskeletal stability [34,43,49,50,67,68] has been demonstrated in various experimental contexts and parallels the structural deterioration observed in irradiated masticatory muscles, where actin–myosin alignment becomes disrupted and mechanical resilience progressively declines [51,52,77]. By preventing actin depolymerization and regulating stress-fiber turnover, *HSP27* may contribute to preservation of muscle integrity under acute stress.

However, under conditions of sustained injury, these same mechanisms have been hypothesized to contribute to maladaptive cytoskeletal reorganization, associated with stiffness, reduced mandibular excursion, and impaired regenerative capacity. Direct evidence for these processes in RIT is currently lacking.

The antioxidant and anti-apoptotic functions of *HSP27* further align with features of chronic radiation injury. In experimental systems, *HSP27* has been shown to support cell survival by maintaining glutathione balance, suppressing caspase-mediated apoptosis, and stabilizing redox-sensitive cytoskeletal proteins [71,72].

While these effects may limit acute cellular injury, prolonged survival of structurally compromised fibroblasts, endothelial cells, and myocytes has been proposed to contribute to persistent fibrosis, microvascular rarefaction, and neuromuscular degeneration [13,51,52,53,54,55,56]. In this context, sustained *HSP27* activation may shift from a protective response toward a contributor to chronic fibro-inflammatory remodeling; however, this transition has not been directly demonstrated in irradiated masticatory tissues.

*HSP27* has also been shown to interact with profibrotic signaling cascades that characterize radiation-induced fibro-atrophy, particularly the *TGF-β* and *NF-κB* pathways [17,29,46,47,48,49,50]. Experimental models of pulmonary, dermal, and cardiac fibrosis demonstrate that *HSP27* participates in myofibroblast differentiation, α-smooth muscle actin expression, fibroblast contractility, and collagen deposition [46,47,48,49,50].

These conserved mechanisms suggest that *HSP27* may influence fibroblast activation and extracellular matrix accumulation within irradiated masticatory muscles; however, extrapolation to RIT remains inferential.

The translational relevance of this framework is further supported by clinical observations indicating that systemic inflammatory, hypoxic, and nutritional biomarkers are associated with RIT risk [62,63,64,65]. These host-response signatures overlap with pathways in which *HSP27* has been implicated, suggesting that *HSP27* expression or phosphorylation status may reflect tissue-level susceptibility to radiation-induced injury. In this integrative model, *HSP27* may link cytoskeletal disruption, oxidative stress, microvascular injury, resistance to apoptosis, and fibroblast activation within a compromised regenerative environment; however, this relationship remains to be validated.

Taken together, the available evidence supports a cohesive hypothesis in which *HSP27* may act as a modulator of radiation-induced tissue remodeling within the masticatory apparatus. However, it must be emphasized that this framework is derived from mechanistic convergence across non-RIT systems and does not establish a direct role for *HSP27* in RIT. Accordingly, these considerations should be interpreted as hypothesis-generating.

Future translational studies should evaluate *HSP27* expression, activation state, and intracellular localization in masticatory tissues before and after radiotherapy, correlate these findings with imaging-defined muscle injury and functional outcomes, and determine whether modulation of *HSP27* signaling influences the development or progression of RIT. If validated, such findings could position *HSP27* as a relevant biomarker or therapeutic target within a broader molecular framework linking radiation-induced cellular stress responses to fibro-neuromuscular degeneration in the masticatory apparatus.

Taken together, these observations suggest that *HSP27* may participate in the interconnected processes of neuromuscular degeneration, microvascular dysfunction, and cytoskeletal remodeling that characterize RIT. However, direct evidence in irradiated masticatory tissues is currently lacking, and these mechanistic parallels should be regarded as hypothesis-generating pending targeted validation.

## 5. Potential Links Between HSP27 and Radiation-Induced Trismus

The preceding sections indicate that RIT arises from a convergence of chronic fibro-atrophic remodeling, neuromuscular degeneration, microvascular compromise, and sustained oxidative–inflammatory stress within the masticatory apparatus [16,17,18,36,37,38,39,40,41,42,43,44,45,46,47,48,49,50,51,52,53,54,55,56,58,77]. *HSP27*, in turn, occupies a central position at the intersection of cytoskeletal regulation, redox homeostasis, apoptosis control, profibrotic signaling, and endothelial resilience [19,20,23,46,47,48,49,50,66,67,68,71,72]. These overlapping biological processes suggest that HSP27 may function as an integrative stress-response mediator within irradiated masticatory tissues. The major biological domains through which *HSP27* may influence susceptibility to RIT are summarized in Table 1. Taken together, these observations support a biologically plausible framework in which *HSP27* contributes to inter-individual variability in the development and severity of RIT, even though direct clinical evidence linking *HSP27* expression to trismus has not yet been established.

From a pathophysiologic standpoint, the fibro-atrophic model of late radiation injury emphasizes persistent activation of transforming growth factor-β (*TGF-β*), fibroblast–myofibroblast transition, excessive extracellular matrix (ECM) deposition, and progressive muscle atrophy as the principal drivers of tissue stiffness and loss of mandibular excursion [16,17,58]. Experimental studies in pulmonary and cardiac fibrosis demonstrate that *HSP27* is upregulated in activated fibroblasts, enhances *TGF-β*-driven myofibroblast differentiation, and promotes fibroblast contractility and collagen synthesis, whereas inhibition of *HSP27* attenuates fibrotic remodeling and epithelial–mesenchymal transition [46,47,48,49,50]. Because irradiated masticatory muscles display imaging and functional characteristics consistent with fibro-atrophy—including MRI evidence of fibrosis, reduced excursion, and progressive tissue stiffness [13,52,53,54,55]—it is reasonable to hypothesize that similar *HSP27*-dependent mechanisms may operate within the local masseter and pterygoid muscle microenvironment.

Skeletal muscle biology further reinforces this potential link. *HSP27* is robustly induced in skeletal muscle in response to contraction-related injury, oxidative stress, disuse, and denervation [32,33,34,35,70,76]. Through phosphorylation-dependent conformational changes, *HSP27* stabilizes actin filaments, regulates stress-fiber dynamics, and protects myofibers from oxidative and mechanical damage [32,33,34,35,43,67,68,76]. However, studies of aging, immobilization, and denervation demonstrate that persistent stress states are accompanied by mitochondrial dysfunction, sustained ROS production, and apoptotic signaling within muscle fibers and motor units [35,46,73,74,75,76,79]. In the context of RIT—where imaging studies demonstrate denervation-like alterations, fascicular disorganization, and progressive atrophy of masticatory muscles [51,52,55,56,77]—chronic *HSP27* activation could permit structurally compromised myocytes and fibroblasts to evade apoptosis [71,72,78], thereby facilitating a gradual transition toward a rigid, fibrotic, and functionally weakened muscle phenotype.

Microvascular and endothelial injury provide an additional mechanistic bridge. Microvascular rarefaction and chronic hypoxia are fundamental features of radiation fibrosis syndrome [16,17,58,59]. *HSP27* plays a critical role in endothelial cytoskeletal stabilization and resistance to oxidative damage, protecting endothelial cells from ROS-mediated apoptosis and preserving barrier integrity under cellular stress [20,50]. In irradiated masticatory muscles—where advanced imaging techniques reveal persistent inflammation, altered perfusion, and microstructural injury [54,55,56,57,58,59,60,61,62,63,64,65,66,67,68,69,70,71,72,73,74,75,76,77]—*HSP27*-mediated survival of injured endothelial and stromal cells could paradoxically promote chronic inflammatory signaling and capillary dysfunction. Over time, such processes may exacerbate hypoxia, perpetuate fibroblast activation, and accelerate myofiber degeneration [16,17,58,76].

Although direct clinical evidence linking *HSP27* expression in masticatory tissues to RIT is currently lacking, several observations provide indirect clinical support for a shared biological framework. *HSP27* is frequently overexpressed in head and neck squamous cell carcinomas—including oral cavity tumors—and has been implicated in resistance to radiotherapy and systemic therapies [24,25,26,30,31]. These findings confirm that tissues of the head and neck region are capable of mounting robust *HSP27* responses to genotoxic and microenvironmental stress. In parallel, recent studies in nasopharyngeal carcinoma cohorts have shown that systemic inflammatory, hypoxic, and nutritional indices—such as Valero’s host index, the Global Immune-Nutrition-Inflammation Index, pan-immune-inflammation value, and hemoglobin-based parameters—strongly predict the development of RIT [3,4,62,63,64]. Because these host factors are closely linked to *NF-κB* activation, oxidative stress, and hypoxia-driven signaling pathways that intersect with *HSP27* biology [23,32,33,66,67,68,71,72,77], it is plausible that *HSP27* functions as a local tissue-level effector within a systemically “pro-trismus” physiological environment.

Collectively, these observations support a testable integrative model. In patients characterized by elevated inflammatory burden, relative hypoxia, and impaired nutritional reserve [3,4,62,63,64], radiotherapy may initiate a sustained stress response within masticatory muscles and their microvasculature. This response involves chronic *ROS* production, mitochondrial dysfunction, and denervation-like injury [13,35,36,37,38,39,40,41,42,43,44,45,46,47,48,49,50,51,52,53,54,55,56,57,58,59,60,61,62,63,64,65,77]. Under these conditions, *HSP27* becomes persistently activated, stabilizing cytoskeletal networks [34,66,68], preventing apoptosis of injured fibroblasts, endothelial cells, and myocytes [71,72,78], and amplifying *TGF-β-* and *NF-κB*-driven profibrotic signaling [17,46,47,48,49,50,58]. Over time, these processes may shift the tissue environment from reversible injury toward entrenched fibro-atrophic remodeling, ultimately manifesting clinically as progressive RIT despite comparable radiation dosimetry.

**Figure 2:**
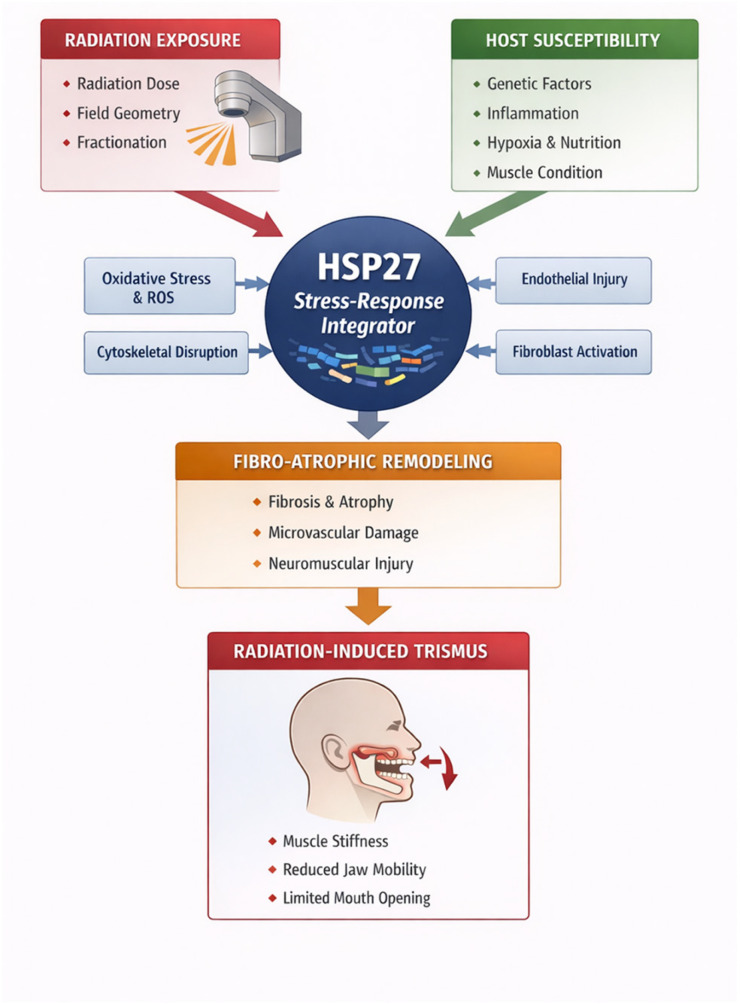
Conceptual model of how *HSP27* may influence individual susceptibility to radiation-induced trismus. Clinical host factors, radiation dose distribution, and tissue stress responses may interact through *HSP27*-regulated pathways involving cytoskeletal stability, redox balance, apoptosis resistance, and fibrosis signaling. Potential translational applications include biomarker development and risk stratification. Created with BioRender.com. https://BioRender.com/3c44flo (accessed on 23 March 2026). Note: Radiotherapy induces oxidative stress, cytoskeletal disruption, endothelial injury, and inflammatory signaling within the masticatory apparatus. These stress responses converge on activation of *HSP27*, a multifunctional small heat shock protein that regulates redox balance, cytoskeletal stability, apoptosis resistance, and fibroblast activation. Acting as a central stress-response integrator, *HSP27* may influence how irradiated tissues adapt to chronic cellular injury. Persistent activation of these pathways promotes fibro-atrophic remodeling characterized by fibrosis, microvascular damage, and neuromuscular degeneration in the masseter and pterygoid muscles. Progressive tissue stiffness and impaired mandibular excursion ultimately lead to the clinical manifestation of RIT. This conceptual overview complements the detailed mechanistic cascade illustrated in Figure 1 and highlights how host susceptibility factors may interact with radiation exposure to shape individual biological responses and variability in RIT risk. The red arrows indicated the maximum mouth opening.

Within this framework, *HSP27* can be conceptualized not merely as a generic stress protein but as a candidate mechanistic mediator and biomarker of RIT. Future investigations in head and neck cancer should therefore (i) quantify *HSP27* expression, phosphorylation status, and subcellular localization in masticatory muscles or relevant surrogate tissues before and after radiotherapy; (ii) correlate these measures with advanced imaging biomarkers of fibrosis, denervation, and microvascular injury [56,57,77]; and (iii) integrate *HSP27*-related parameters with systemic inflammatory, hypoxic, and nutritional indices [3,4,62,63,64,65] within multivariable models of RIT susceptibility. Complementary preclinical studies employing *HSP27* inhibition or genetic modulation in skeletal muscle and fibrosis models [46,47,48,49,50] may further clarify whether *HSP27* represents a viable therapeutic target for preventing or mitigating RIT. Although direct clinical validation in RIT is currently lacking, these convergent mechanistic and translational data support investigation of *HSP27* as a candidate biomarker or mediator of inter-individual susceptibility. Through such investigations, the proposed *HSP27–RIT* relationship could evolve from a biologically plausible hypothesis into a clinically actionable framework for risk stratification and intervention (Figure 2).

## 6. Translational Implications and Future Directions

The integrative framework developed in this review positions *HSP27* as a plausible mediator and potential biomarker of RIT. Throughout this review, mechanistic interpretations have been explicitly derived from non-RIT biological systems, including fibrosis models, radiation injury studies, and skeletal muscle research, and are therefore intended to generate hypotheses rather than establish causal relationships in RIT. Although direct evidence linking *HSP27* to RIT remains limited, the convergence of mechanistic insights from fibrosis biology, skeletal muscle stress responses, endothelial injury, and neuromuscular degeneration provides a compelling rationale for systematic translational investigation. Advancing *HSP27* from a conceptual hypothesis to a clinically actionable biomarker or therapeutic target will require coordinated research spanning biomarker discovery, mechanistic modeling, and early-phase therapeutic exploration.

A first priority is to determine whether *HSP27*-related biomarkers provide predictive information beyond conventional dosimetric parameters and systemic host-response indices. Because susceptibility to RIT appears to arise from complex interactions between local radiation injury and systemic physiological states—including inflammation, hypoxia, and nutritional reserve—assessment of baseline and treatment-induced *HSP27* expression, phosphorylation status, and intracellular localization may offer novel predictive insights. These measurements could be obtained from tumor specimens, adjacent mucosa, circulating extracellular vesicles, or peripheral blood cells. Assessment of pretreatment circulating or plasma *HSP27* levels may be particularly attractive as a minimally invasive strategy for baseline risk stratification. Integrative models combining *HSP27*-related biomarkers with radiation dosimetry and established systemic inflammatory or nutritional indices may further improve individualized prediction of RIT susceptibility. Correlation of *HSP27* dynamics with advanced imaging biomarkers—such as MRI- and diffusion-based signatures of fibrosis, denervation, and chronic inflammation—would help clarify whether *HSP27* activity tracks with the structural evolution of injury within the masticatory apparatus.

A second translational priority involves developing mechanistically grounded preclinical models that directly interrogate the role of *HSP27* in radiation-induced fibro-atrophic remodeling. Such models should replicate the biomechanical and metabolic stresses characteristic of the masseter and pterygoid muscles following radiotherapy, allowing controlled manipulation of *HSP27* expression or phosphorylation. These experiments would determine whether *HSP27* activation primarily functions as a protective stress response or contributes to maladaptive fibrosis, neuromuscular degeneration, and microvascular dysfunction. Establishing this distinction is essential before considering therapeutic modulation of *HSP27* pathways.

A further implication of this framework is the potential for early-phase interventional strategies targeting *HSP27*-regulated pathways. Because *HSP27* influences oxidative stress responses, *TGF-β* signaling, fibroblast activation, cytoskeletal remodeling, and resistance to apoptosis, pharmacologic inhibitors of *HSP27* or modulators of the *p38–MAPKAPK2* signaling axis could eventually be evaluated as adjunctive approaches for patients identified as having elevated biological susceptibility to RIT. Any such strategies must be approached cautiously, however, as *HSP27* also contributes to physiological stress tolerance and neuromuscular stability in normal tissues. Interpretation of *HSP27* as a therapeutic target should therefore be cautious, as inhibition of a cytoprotective stress-response pathway could theoretically impair normal tissue recovery in some contexts. Careful determination of therapeutic timing—whether prophylactic, concurrent with radiotherapy, or directed at early stages of fibro-atrophic remodeling—will therefore be critical to balance potential benefits and risks.

Ultimately, progress in this area will depend on integrated longitudinal studies that combine radiation dosimetry, advanced imaging, systemic inflammatory and nutritional biomarkers, and molecular measures of *HSP27* activity. Linking these domains within unified predictive frameworks may clarify whether *HSP27* meaningfully contributes to inter-individual variability in RIT susceptibility and whether it warrants incorporation into risk-stratified prevention strategies. Insights from such work could also guide the design of interventional trials evaluating intensified anti-fibrotic therapies, individualized physiotherapy protocols, or redox-modulating strategies informed by *HSP27* biology.

In summary, while definitive clinical evidence remains to be established, the mechanistic coherence of the *HSP27*–RIT hypothesis supports a structured translational research agenda. By bridging molecular stress-response pathways with imaging biomarkers, functional outcomes, and therapeutic opportunities, future studies may determine whether *HSP27* can evolve from a theoretical construct into a measurable biomarker and a potential target for personalized strategies aimed at preventing or mitigating RIT. Collectively, the available evidence supports a conceptual model in which *HSP27* functions as a molecular integrator of oxidative stress, cytoskeletal disruption, and fibro-atrophic remodeling in irradiated masticatory tissues, thereby potentially shaping individual susceptibility to RIT.

## 7. Conclusions and Future Perspectives

RIT remains a complex and incompletely resolved late toxicity of head and neck radiotherapy, driven by the interplay of fibrosis, neuromuscular degeneration, microvascular injury, chronic oxidative stress, and impaired tissue repair within the masticatory apparatus. The evidence synthesized throughout this review highlights that *HSP27* may occupy a biologically strategic position at the intersection of these processes. Its core functions—regulation of actin dynamics, modulation of redox balance, inhibition of apoptosis, and facilitation of fibroblast activation—map closely onto the structural and functional deterioration observed in irradiated masticatory muscles.

Current evidence remains insufficient to define whether *HSP27* functions principally as a biomarker of tissue vulnerability, a mechanistic driver of fibro-neuromuscular injury, or a secondary epiphenomenon of broader radiation damage. Existing findings are heterogeneous and strongly context-dependent, underscoring the need for prospective clinical cohorts and tissue-specific experimental models. Resolving this distinction will be essential not only for advancing mechanistic understanding, but also for determining whether *HSP27* has meaningful clinical utility in risk stratification, prevention, or targeted intervention strategies for RIT.

Future research should integrate molecular analyses of *HSP27* expression, phosphorylation, and intracellular localization with advanced imaging signatures of fibrosis and neuromuscular injury, ideally within longitudinal clinical cohorts of patients undergoing head-and-neck radiotherapy. Parallel efforts using mechanistically informed preclinical models—including targeted genetic or pharmacologic modulation of *HSP27*—will be necessary to elucidate causal relationships and define the therapeutic potential, as well as the biological limitations, of *HSP27*-directed interventions. Ultimately, strategies that combine biological susceptibility markers with optimized radiotherapy planning and early rehabilitative interventions may offer the most promising path toward personalized prevention of RIT.

As the field progresses, *HSP27* has the potential to evolve from a conceptual mediator into a practical tool for biological risk stratification, mechanistic insight, and potentially therapeutic innovation. Integrating molecular stress-response biology with advances in imaging, dosimetry, and precision radiotherapy may substantially improve our ability to predict, prevent, and manage RIT. The principal contribution of this review is the proposal of a clinically anchored, testable framework linking *HSP27* biology to susceptibility and progression of RIT. While current evidence does not establish a direct role for *HSP27* in RIT, the convergence of mechanistic pathways across radiation biology, fibrosis, and skeletal muscle adaptation provides a biologically plausible rationale for future investigation. Prospective clinical studies and tissue-specific experimental models are required to determine whether *HSP27* functions primarily as a biomarker, mechanistic mediator, or epiphenomenon in RIT. Until such data are available, the proposed framework should be interpreted as hypothesis-generating rather than confirmatory.

## Figures and Tables

**Table 1:** Mechanistic Evidence Linking *HSP27*-Regulated Cellular Stress Pathways to the Pathogenesis of Radiation-Induced Trismus.

Biological Domain	Evidence from HSP27 Research	Relevance to Radiation-Induced Trismus (RIT)	Key References
Oxidative stress regulation	HSP27 maintains glutathione homeostasis and protects proteins from ROS-mediated damage	Chronic ROS production is a hallmark of irradiated muscle and contributes to fibrosis and myofiber degeneration	[35,71,76]
Cytoskeletal stabilization	Phosphorylated HSP27 regulates actin polymerization and stress-fiber organization	Cytoskeletal integrity is critical for maintaining muscle elasticity and mandibular mobility	[34,43,67,68]
Apoptosis inhibition	HSP27 blocks cytochrome-c release and caspase-3 activation	Survival of damaged fibroblasts and myocytes may promote persistent fibro-atrophic remodeling	[71,72]
Fibroblast activation and fibrosis	HSP27 enhances TGF-β signaling and supports myofibroblast differentiation	Fibroblast activation drives extracellular matrix deposition and progressive muscle stiffness	[46,47,48,49,50]
Inflammatory signaling	HSP27 interacts with NF-κB and p38 MAPK pathways regulating cytokine responses	Chronic inflammatory signaling contributes to long-term radiation tissue injury	[66,67]
Endothelial resilience	HSP27 stabilizes endothelial cytoskeleton and protects against oxidative apoptosis	Endothelial dysfunction and microvascular rarefaction are central to radiation fibrosis	[20,50]
Neuromuscular stress response	HSP27 is induced by denervation, mechanical strain, and oxidative muscle injury	Denervation-like changes and muscle atrophy are common in irradiated masticatory muscles	[70,71,72,73,74,75,76,78]
Integrative stress-response signaling	HSP27 integrates oxidative, cytoskeletal, inflammatory, and apoptotic pathways during cellular stress	May function as a molecular mediator linking radiation-induced tissue injury to fibro-neuromuscular degeneration and RIT development	This review

## Data Availability

No new data were created or analyzed in this study. Data sharing is not applicable to this article.
